# Non-linear laws of echoic memory and auditory change detection in humans

**DOI:** 10.1186/1471-2202-11-80

**Published:** 2010-07-03

**Authors:** Koji Inui, Tomokazu Urakawa, Koya Yamashiro, Naofumi Otsuru, Makoto Nishihara, Yasuyuki Takeshima, Sumru Keceli, Ryusuke Kakigi

**Affiliations:** 1 Department of Integrative Physiology, National Institute for Physiological Sciences, Myodaiji, Okazaki 444-8585, Japan; 2 Multidisciplinary Pain Center, Aichi Medical University, Aichi 480-1195, Japan

## Abstract

**Background:**

The detection of any abrupt change in the environment is important to survival. Since memory of preceding sensory conditions is necessary for detecting changes, such a change-detection system relates closely to the memory system. Here we used an auditory change-related N1 subcomponent (change-N1) of event-related brain potentials to investigate cortical mechanisms underlying change detection and echoic memory.

**Results:**

Change-N1 was elicited by a simple paradigm with two tones, a standard followed by a deviant, while subjects watched a silent movie. The amplitude of change-N1 elicited by a fixed sound pressure deviance (70 dB vs. 75 dB) was negatively correlated with the logarithm of the interval between the standard sound and deviant sound (1, 10, 100, or 1000 ms), while positively correlated with the logarithm of the duration of the standard sound (25, 100, 500, or 1000 ms). The amplitude of change-N1 elicited by a deviance in sound pressure, sound frequency, and sound location was correlated with the logarithm of the magnitude of physical differences between the standard and deviant sounds.

**Conclusions:**

The present findings suggest that temporal representation of echoic memory is non-linear and Weber-Fechner law holds for the automatic cortical response to sound changes within a suprathreshold range. Since the present results show that the behavior of echoic memory can be understood through change-N1, change-N1 would be a useful tool to investigate memory systems.

## Background

Immediate detection of an abrupt change in the environment is one of the most important functions of sensory systems. Actually, neural networks sensitive to sensory changes are known in humans [1,2]. If a change-detection system operates spontaneously to orient the individual to the new condition, investigating the preattentive activation process in the brain in response to sensory changes can help us to understand the mechanisms of this sensory change-detection system. Change-detection process necessitates the comparison of the new event with the preceding condition, and therefore, should involve sensory memory, i.e. the ability to hold sensory information in a readily accessible state temporarily [3-6]. In the auditory system, the mechanism of change-detection and/or its relation to the memory system has been studied using mismatch negativity (MMN) [5,7-10] and change-N1, a subcomponent of N1 [11-15]. MMN is an electromagnetic response to a discriminable change in any regular aspect of auditory stimulation, which usually peaks 150 to 200 ms following the onset of the change. MMN is commonly obtained under a so-called oddball paradigm, in which a deviant stimulus is interspersed among a frequently presented standard stimulus. Although MMN is a component of event-related brain potentials, the mismatch response has been recorded also using other methods including magnetoencephalography (MEG) [16-20], positron emission tomography [21,22], functional magnetic resonance imaging [23,24], optic imaging [25], and intracranial recordings [26,27]. A commonly accepted interpretation of MMN is that it is generated by an automatic change-detection process in which a disconcordance is found between the input from the deviant auditory event and the sensory-memory-representation of the regular aspects of the preceding auditory stimulation [5,7] (for a different interpretation, see [28]). Therefore, brain activity in response to sensory changes is expected to be affected by a memory trace of a preceding event (memory storage), the time between the new and the preceding events (memory decay), and the degree of physical difference between the two events. In fact, previous studies show that the latency and amplitude of MMN is affected by the interval between the standard and deviant [17,19,29,30] and magnitude of deviance [31-35] (but see [20]).

Change-N1, which is elicited by a sudden change in a continuous tone and peaks approximately 100 ms after the onset of the change, has been also used to investigate higher auditory processes [11-15]. The scalp distribution of change-N1 differs from that of N1 evoked at the onset of the tone, suggesting that the neural populations involved in these responses are at least partially different from one another. Although change-N1 differs from MMN in that MMN does not contain the N1 component, change-N1 is also considered to relate to an auditory store [11-15].

Change detection has long been studied in psychophysics using a sensory threshold. Weber [36] first pointed out that for the tactile system, the ratio of a just noticeable difference (JND) in intensity to the physical variable being compared (ΔI/I) is constant regardless of variations in I. Fechner [37] showed that internal, intensive scales can be reconstructed after assuming that the ratio ΔI/I represents a unitary value in sensation (ΔS) and integrated the function ΔS = k * ΔI/I, which led to the famous, logarithmic law of subjective intensity (Weber-Fechner law). Although this resulted in great progress in the psychophysic study of the subjective internal world (for recent review, see [38]), it remains to be elucidated whether the phenomenon seen using threshold measurements applies to suprathreshold situations [39] or whether this law can be used to scale the magnitude of sensed differences. Furthermore, whether these psychological laws hold for brain responses in humans is yet to be clarified.

In this study, we investigated the relationship between cortical responses and the magnitude of differences in various stimulus parameters by using the change-N1 component in the auditory system that serves as an indicator of the brain's change-detection system. Change-N1 was also expected to provide clues about echoic memory that is auditory equivalent of sensory memory. Since change detection has been shown to be affected by the timing of the deviant stimulus during the standard-deviant sequence (i.e. status of the auditory memory trace), at first, we changed the interval between the standard and deviant stimuli to examine change detection and echoic memory decay through the change-N1 response (Experiment 1) and then, changed the duration of the standard stimulus to examine echoic memory storage (Experiment 2) using two tones of different sound pressure (70 vs 75 dB) under a simple even probability paradigm. In Experiment 3, we examined effects of the magnitude of the deviance using changes in repetitive brief tones.

## Results

### Experiment 1

**Figure 1:**
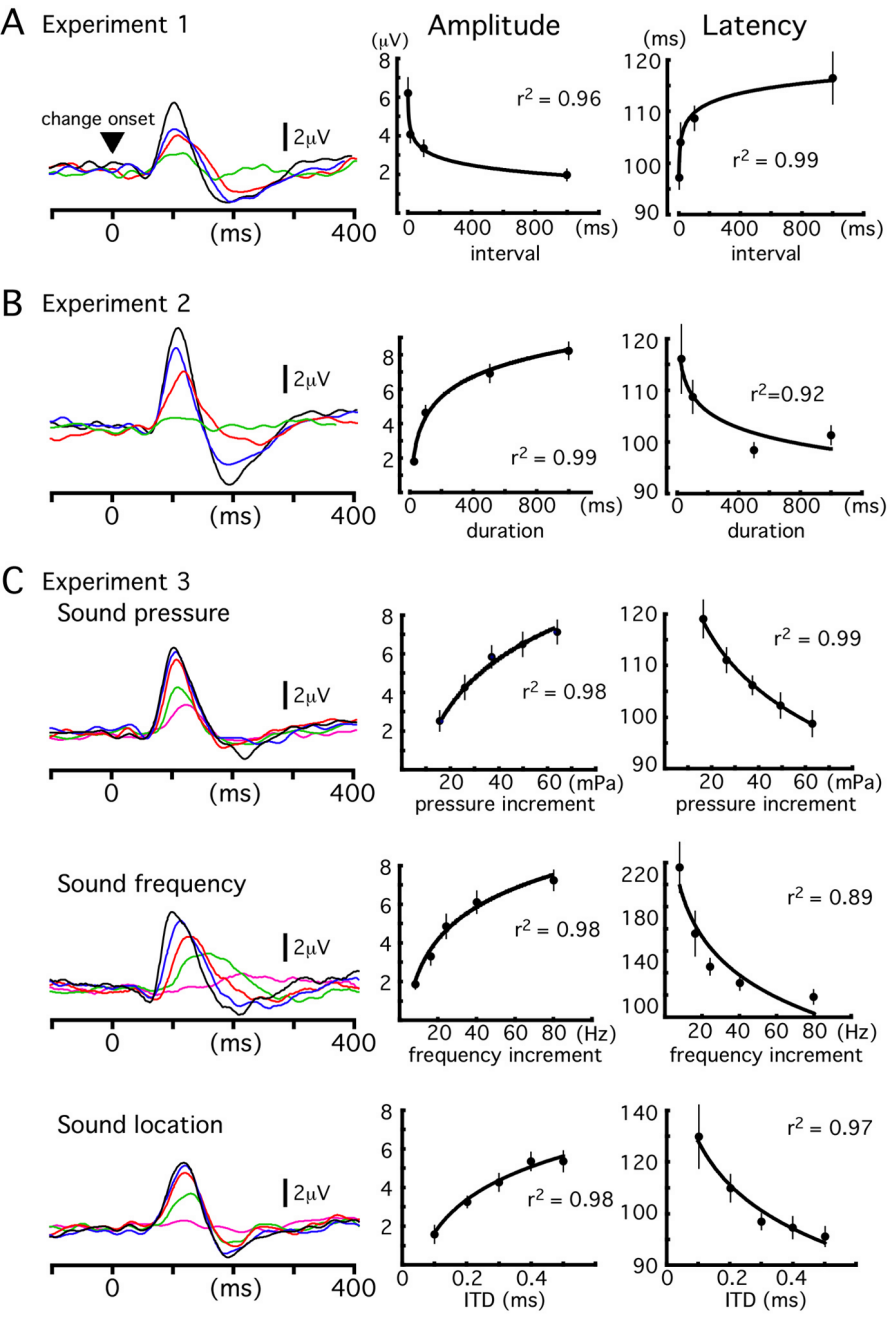
**Grand-averaged waveforms of change-N1 across seven subjects and the peak amplitude and latency of change-N1**. A, effects of the interval between the standard and deviant sounds. Four intervals, 1, 10, 100, and 1000 ms, were tested. B, effects of the duration of the standard sound. Four durations, 25, 100, 500 and 1000 ms, were tested. C, effects of the magnitude of deviation on change-N1. Sound pressure (70 dB vs. 72, 73, 74, 75 and 76 dB), sound frequency (800 Hz vs. 808, 816, 824, 840 and 880 Hz), and sound location (interaural time delay, ITD, of 0.1, 0.2, 0.3, 0.4 and 0.5 ms) deviations were tested. The mean amplitude and latency across subjects of each experiment are plotted against the degree of the deviation of each variable. Error bars indicate ± SE. The correlation coefficient, r^2^, in this figure shows values calculated with a logarithmic function using the mean data. Corresponding values for individual data appear in the text.

**Figure 2:**
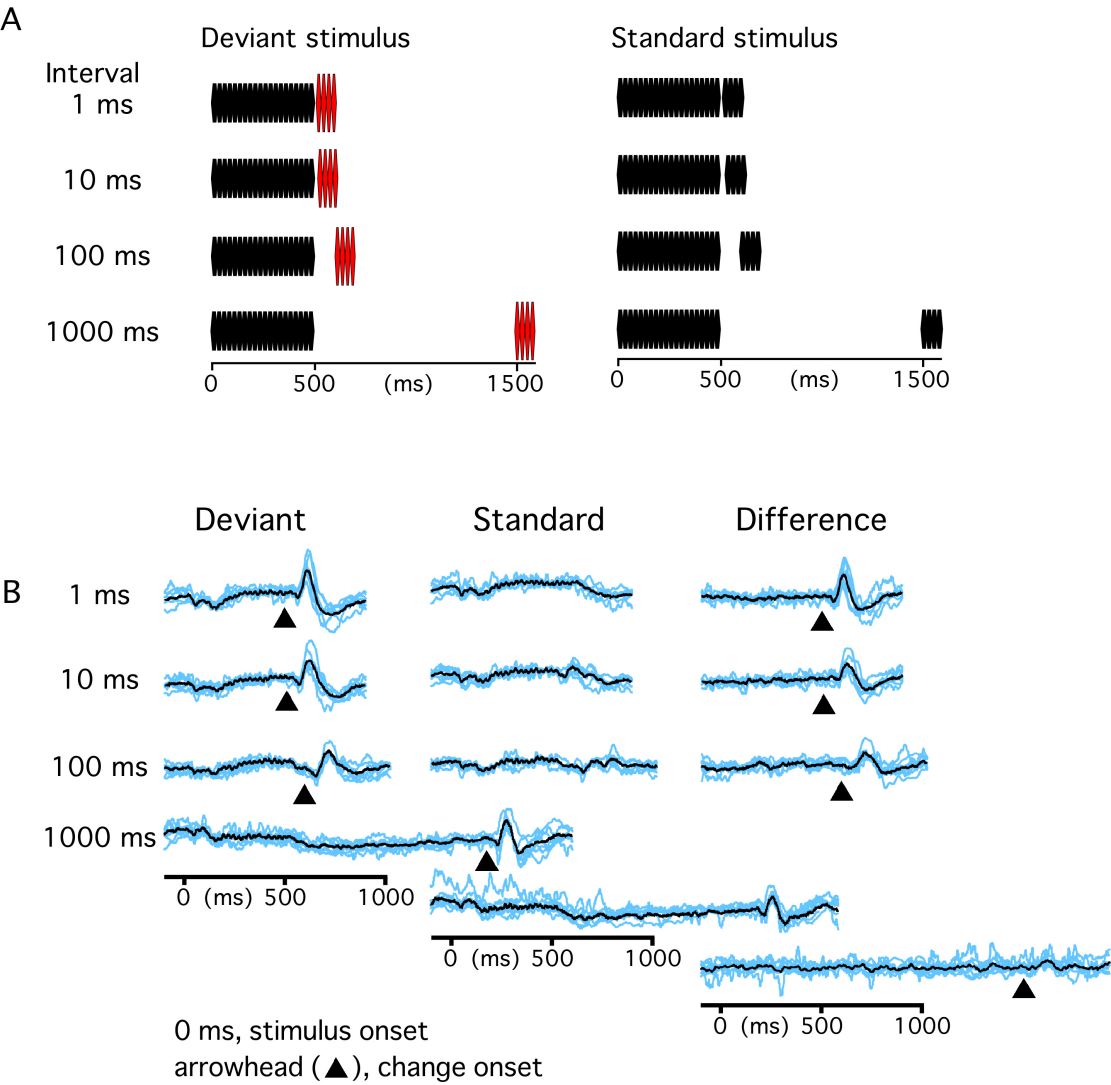
**Effects of the interval between the standard sound (70 dB) and deviant sound (75 dB) on the deviant sound-induced change-N1**. A, experimental paradigm. B, evoked brain potentials of all subjects (blue lines) and their grand-averages (black).

Effects of the interval between the standard sound and deviant sound on change-N1 were examined by varying the interval between two stimuli from 1 to 1000 ms. Results of each experiment are summarized in Fig. 1. Original and difference waveforms of all the subjects are shown in Fig. 2. The deviant sound elicited a clear component at around 100 ms following its onset. We refer to this component as change-N1 in this paper. The mean peak latency and amplitude of change-N1 for each condition are listed in Table 1. Results show that the amplitude of change-N1 decreased as the interval between the standard and deviant stimuli increased. The amplitude was negatively correlated with the logarithm (correlation coefficient, r^2 ^= 0.96) or power (r^2 ^= 0.97) of the interval for averaged data (Fig. 1A). In individual subjects, the r^2 ^of 0.94 ± 0.06 for the logarithmic function or 0.95 ± 0.03 for the power function was clearly larger than that for the linear function (0.58 ± 0.17). The peak latency of change-N1 increased with an increase in the interval with a logarithmic function for the averaged data (r^2 ^= 0.99). However, for individual data, results of the curve fitting usually did not reach a significant level for either the logarithmic (r^2 ^= 0.54 ± 0.25), power (0.54 ± 0.25), exponential (0.61 ± 0.35), or linear (0.59 ± 0.33) function.

**Table 1:** Mean peak latency and amplitude of change-N1.

Experiment 1	1		10	100	1000 (ms)_n = 6_
Amplitude (μV)	6.2 (2.4)		4.1 (1.4)	3.4 (1.4)	2.0 (1.0)

Latency (ms)	97 (6)		104 (10)	109 (6)	117 (13)

Experiment 2	25		100	500	1000 (ms)

Amplitude	1.8 (0.6)		4.6 (1.3)	6.9 (1.6)	8.3 (1.6)

Latency	116 (18)		109 (9)	99 (4)	101 (5)

Experiment 3					

Pressure	72_n = 6_	73	74	75	76 dB

Amplitude	2.5 (1.3)	4.3 (1.4)	5.9 (1.6)	6.5 (1.6)	7.1 (1.6)

Latency	119 (10)	111 (7)	106 (5)	102 (7)	99 (7)

Frequency	808	816	824	840	880 (Hz)

Amplitude	1.9 (0.9)	3.3 (1.6)	4.9 (2.2)	6.1 (2.0)	7.3 (1.8)

Latency	216 (31)	156 (28)	126 (11)	112 (9)	99 (10)

Location	0.1_n = 5_	0.2	0.3	0.4	0.5 (ms)

Amplitude	1.6 (1.1)	3.4 (0.9)	4.3 (1.3)	5.4 (1.3)	5.4 (1.5)

Latency	135 (33)	125 (15)	119 (9)	117 (12)	116 (11)

Data are expressed as the mean (SD)

### Experiment 2

**Figure 3:**
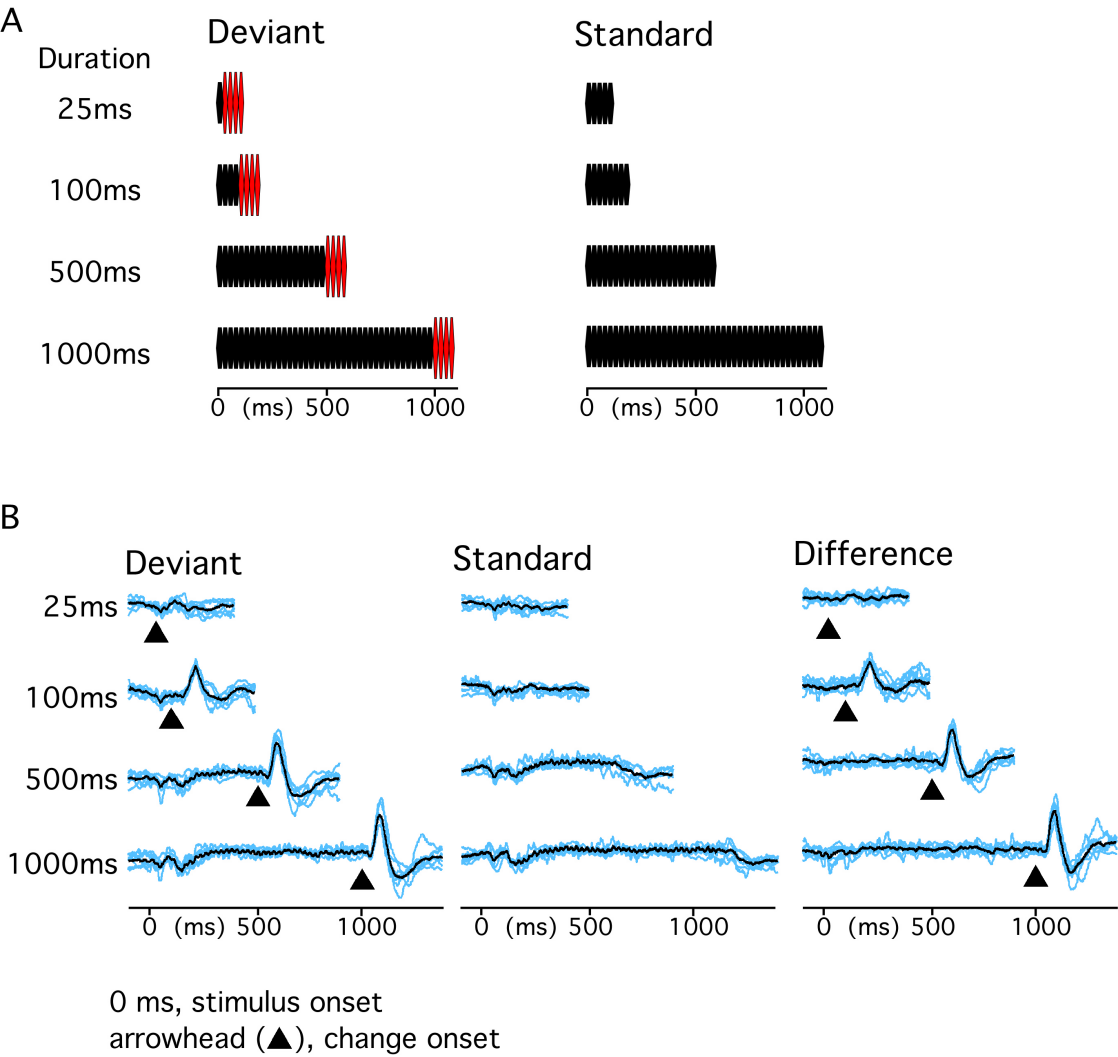
**Effects of the duration of the standard sound (70 dB) on the deviant sound (75 dB)-induced change-N1**. A, experimental paradigm. B, evoked brain responses of all the seven subjects (blue lines) and their grand-averages (black).

We examined the effects of the duration of the standard sound. The results described above led us to expect that a longer standard sound would increase the memory storage for itself and consequently the deviant sound would evoke a larger change-N1. Results were as expected. The amplitude of change-N1 as a function of the duration of the standard sound showed a positively accelerated curve (Figs. 1B and 3). Results of the curve fitting of individual data showed that the r^2 ^value was largest for the logarithmic function (0.95 ± 0.04) followed by the power (0.92 ± 0.07), exponential (0.84 ± 0.14), and linear (0.81 ± 0.12) functions. The peak latency tended to decrease with an increase in the duration in all the subjects. However as in Experiment 1, the curve fitting in individual subjects was sometimes difficult (r^2 ^= 0.8 ± 0.34 for exponential, 0.68 ± 0.28 for logarithmic, 0.68 ± 0.28 for power, and 0.56 ± 0.31 for linear).

### Experiment 3

**Figure 4:**
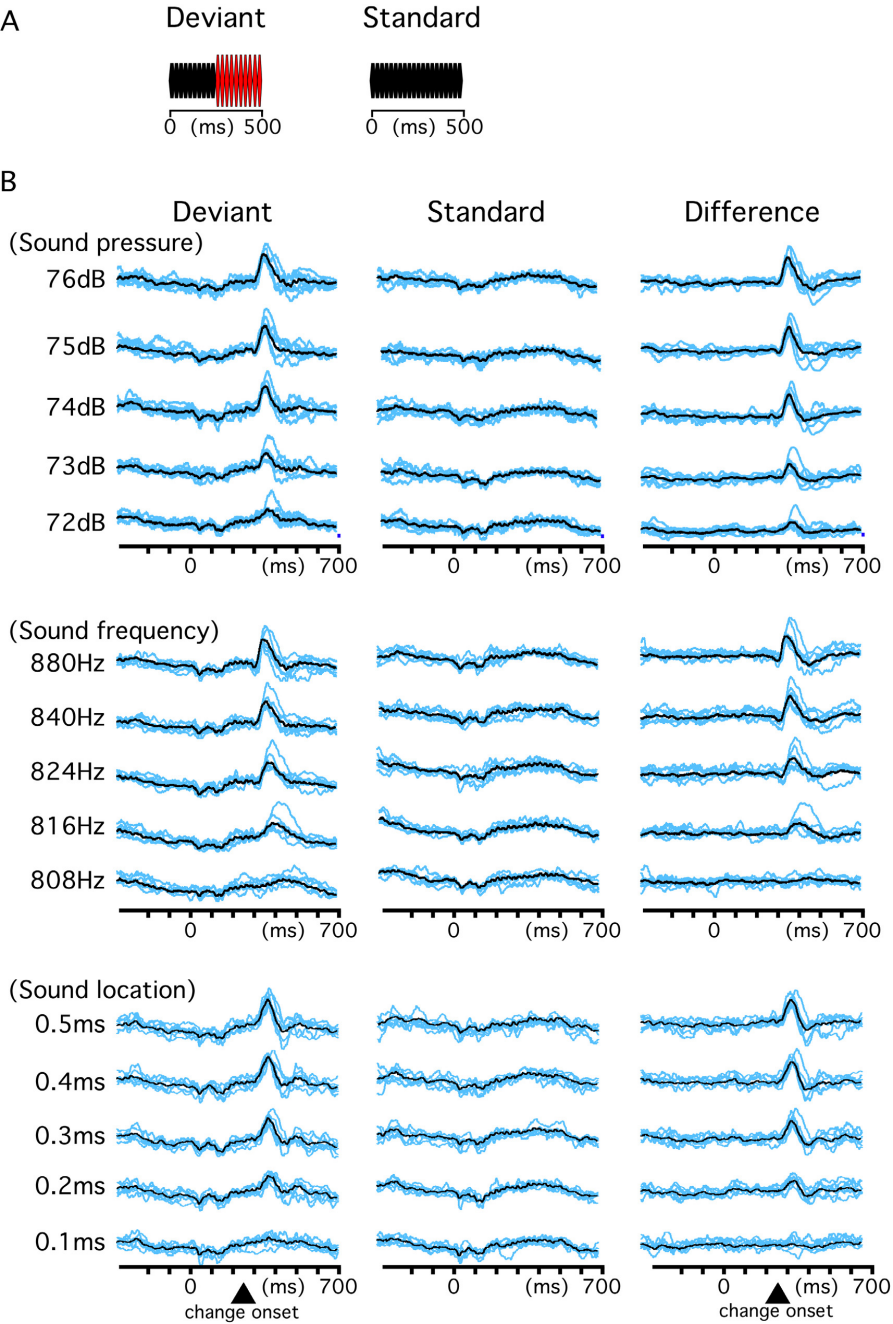
**Effects of the magnitude of the physical difference between the standard and deviant sounds on change-N1**. A, stimulation paradigm. B, evoked waveforms of all the subjects (blue) and their grand-averages (black).

Given that several features of a sound can be stored in echoic memory [40] or a long auditory store [3] and the generation of change-N1 depends on a memory-comparison between the new event and preceding state, the degree of the physical difference between the standard and deviant sounds should affect the amplitude of change-N1. We tested this using three variables: sound pressure, sound frequency, and sound location. Results are shown in Figs. 1C and 4. For all three variables tested, the relationship between the amplitude of change-N1 and the magnitude of physical differences between the standard and deviant sounds was not linear. In general, the relationship was explained best by the logarithmic function (r^2 ^= 0.94 ± 0.04 for sound pressure, 0.94 ± 0.04 for sound frequency, and 0.91 ± 0.1 for sound location) and worst by the linear function (0.87 ± 0.08, 0.82 ± 0.13 and 0.85 ± 0.13). The latency of change-N1 decreased with an increase in the magnitude of the deviance. The r^2 ^value of the curve fitting was largest for the exponential (0.91 ± 0.1, 0.97 ± 0.03 and 0.75 ± 0.19) and smallest for the linear (0.73 ± 0.14, 0.61 ± 0.09 and 0.56 ± 0.17) functions. In all three experiments (Experiments 1, 2 and 3), there was a tendency for the amplitude to follow a logarithmic function and for latency to follow an exponential function.

## Discussion

The present results suggest that change-N1 is a product of an automatic change-detection system that receives auditory information processed in earlier cortical areas and generates change-specific signals proportional to the magnitude of the deviance, at least under the present experimental conditions. Results of Experiment 3 showed that Weber-Fechner law holds for the automatic cortical response to auditory changes within a suprathreshold range. One weakness of Fechner's formulation is that there is no known relationship between the size of a JND and the rate of growth of subjective magnitude [41]. The present results suggest that when the magnitude of a perceived difference is applied to Weber's law, Weber-Fechner law might hold for the magnitude of the perceived differences within a suprathreshold range. That is, the magnitude of the perceived difference would be logarithmically related to the degree of the difference between the two stimuli. Although the present study did not provide psychological data, there is a psychophysics study supporting this view [39]. In terms of the survival of animals, such a non-linear function would work well within a physiologically significant range of sensory changes. In this regard, a limitation of Weber's law, that just noticeable differences are constant only within a certain range of sensory intensities, seems reasonable. When the stimulus or the difference between a new event and the preceding state is strong enough to fully orient to the new event, a further increase in brain activity specific to change detection would be unnecessary.

The results of Experiment 1, that a decay of echoic memory has a non-linear function in time, are consistent with psychological studies using a dichotic listening task showing that recall performance decreases with time in a typical negatively accelerated fashion [42,43]. Some previous ERP and MEG studies also showed a decrease in the amplitude of mismatch responses with an increase in the interstimulus interval [17,19,29]. In addition to this, the present study showed that echoic memory has a non-linear storage function in time. Although whether this rule can be applied to other more complicated memory systems is unclear, these results might show one fundamental mechanism of memory. Echoic memory shares features with short-term memory or working memory [6]. Since the present results show that the behavior of echoic memory can be understood through change-N1, change-N1 would be a useful tool to investigate memory systems in addition to psychological methods. However, there remains the possibility that the present results reflect more than one form of memory. There are several lines of evidence that support the existence of two different auditory stores, short and long [3]. The short form refers to a literal store that decays within one second of a stimulus. For example, psychological studies using auditory persistence paradigms [44-47], integration phenomena of sound intensity and time [48], and the shift of the auditory threshold [49] showed a short store of 200 to 300 ms. A long auditory store retains a spectro-temporal structure of sounds over several seconds or more and thus differs from the shorter type by its storage properties and temporal span. Psychological studies, for example using auditory modality superiority effects [50], dichotic listening [43], or two-stimulus comparisons [51] have provided data supporting the existence of this type of store. Therefore, these two different auditory stores might be involved in the generation of the change-N1 component in the present study. There is a possibility that the time course of change-N1 in Experiments 1 and 2 comes from two overlapping curves.

The main component of auditory evoked potentials peaking at around 100 ms (N1 or N100) is known to have several subcomponents (for review, see [52]). The present study using only one exploring electrode could not clarify the composition of change-N1. However, although the present study did not employ a specific stimulation paradigm, change-N1 resembles MMN in several aspects: 1) a frontocentral negative and mastoid-positive field distribution, 2) independence of attention [8], 3) positive relation of the peak amplitude and inverse relation of the peak latency to the magnitude of the difference between the standard and deviant [31,33,35,53], and 4) dependence on the memory trace [5,54]. This suggests that change-N1 might contain the MMN component at least in part or these two have a similar, though not identical, generating mechanism. However, as for the amplitude of MMN elicited by sound frequency deviance, Horváth *et al. *[55] found no effects of the magnitude of deviance on it. One possible reason for the discrepancy with the present results is their subtraction method used to obtain MMN. For the control condition, they presented all the deviant stimuli randomly at the same probability under an oddball sequence equal to that used in the standard-deviant condition. Then they used the deviant-control difference waveform rather than deviant-standard difference to obtain the 'genuine' MMN, which can eliminate the contribution of the N100 component to the difference waveform [56,57]. As we did not use a subtraction procedure, change-N1 in the present study could contain the N100 component. In a study using MEG, Lavikainen *et al. *[58] found that a change in frequency of a continuous tone produced magnetic responses consisting of two separable components, which probably correspond to N100 and MMN. Therefore, the discrepancy between Horváth's findings and the present findings might suggest that the relationship between the amplitude of the response and the magnitude of deviance is due to the change-sensitive N1 subcomponent and not MMN.

Along with the frequency deviance-elicited MMN [57], Schröger's group proposed a method of eliminating possible contamination by N100 [59] and obtaining a 'genuine' MMN for studying sound location [56] and sound pressure [60] deviances. This is important because it has been argued that N100 and MMN reflect different cognitive processes in audition [61-63] (for review, see [7]), while some investigators proposed that MMN is part of a modulated N100 response, and therefore, there is no genuine MMN [64] (for review, see [28]), that is, MMN is the difference between the adapted N100 elicited by a standard and non-adapted N100 elicited by a deviant (adaptation model). However, as for the long-lasting debate on N100 and MMN in general, the present findings neither support nor rule out the adaptation model or memory-based model. This matter is beyond the scope of the present study. Studies comparing the 'genuine' MMN and change-N1 might help to resolve this issue.

It remains to be clarified whether the gradual change of the response obtained in this study is present in each trial for each subject, or observed due to the averaging across trials and/or due to the different thresholds for the change in different participants while the phenomenon actually responds in an all-or-none manner (see [30]). In the present study, the curve fitting for the individual subjects was difficult with any model for latency. Although it appears that results of analyses of individual data support the idea that both the amplitude and latency of change-N1 behave in a non-linear fashion, no definite conclusion was reached regarding the manner of the behavior, for example, logarithmic or exponential like in psychological studies where there are numerous discussions of the model to explain sensory perception [65]. As for the possibility that the amplitude of the averaged response across trials is not determined by the amplitude of the response in each trial, Atienza *et al. *[66] showed in a unique study investigating sleep-dependent enhancement that the enhancement of postsleep MMN amplitude was a result of a reduction in the MMN latency jitter rather than an increase in amplitude. This issue remains to be resolved.

It is suggested that MMN could be used to determine the degree of abnormality in auditory perception, attention, and memory, and in fact, previous studies have found an attenuated or delayed MMN in clinical disorders such as schizophrenia [67]. We believe that the stimulation paradigm used in this study will improve the method for replicable recordings of the change-related response in individual patients, and in addition, for separate evaluations of discrimination accuracy, memory establishment, and memory decay. Since the present results were obtained in only seven subjects whose age varied considerably, normative data should be collected in a study with a larger sample.

## Conclusions

The present findings suggest that temporal representation of echoic memory is non-linear and Weber-Fechner law holds for the automatic cortical response to sound changes within a suprathreshold range. Although whether this rule can be applied to other more complicated memory systems is unclear, these results might show one fundamental mechanism of memory. Since the behavior of echoic memory can be understood through change-N1, change-N1 would be a useful tool to investigate memory systems.

## Methods

The experiment was performed on seven (one female and six male) healthy right-handed volunteers, aged 26-45 (31 ± 7) years. The study was approved in advance by the Ethics Committee of the National Institute for Physiological Sciences, Okazaki, Japan, and written consent was obtained from all the subjects.

### Stimulation

**Figure 5:**
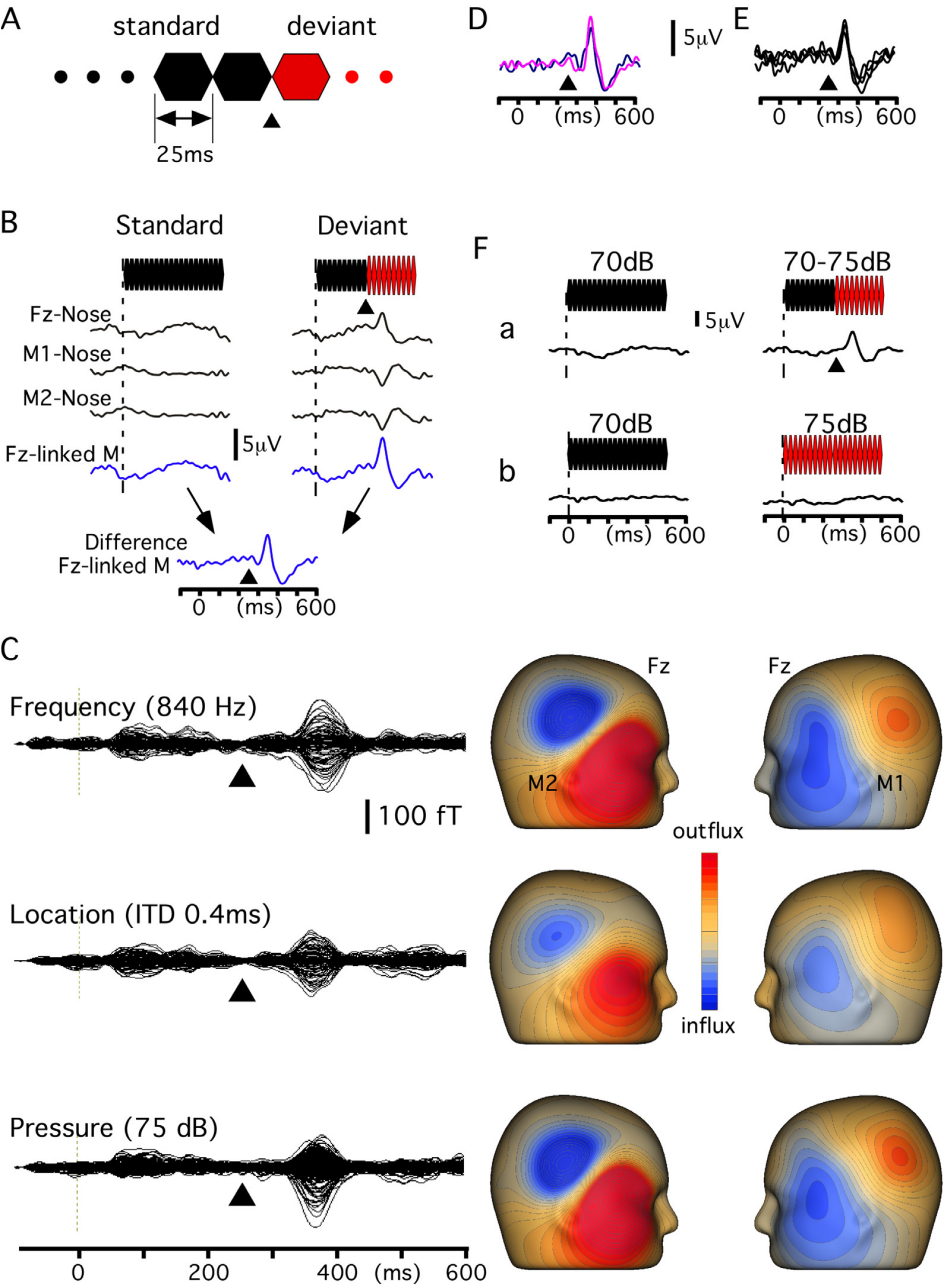
**Change-N1 of event-related potentials**. A, the sound stimulus with an abrupt change used in this study. B, brain potentials recorded at Fz and mastoids produced by the Standard stimulus with a 500-ms standard sound (800 Hz, 70 dB SPL) and Deviant stimulus with a 250-ms standard followed by a 250-ms deviant (75 dB). C, Magnetic fields evoked by auditory deviations. Left, superimposed waveforms obtained from 102 magnetometers (Vectorview; ELEKTA Neuromag, Heksinki, Finland) elicited by three deviant stimuli. The Standard stimulus was a 500-ms standard sound (800 Hz at 70 dB). The Deviant stimulus was a 250-ms standard sound followed without a blank by a 250-ms deviant sound (840 Hz, ITD 0.4 ms or 75 dB). Right, magnetic field distribution at the peak of change-N1. Deviant stimuli evoked a magnetic field response consistent with symmetric dipoles with an intracellular current directed to the mastoid. These dipoles in both hemispheres are expected to create a positivity at both mastoids and a larger negativity (summation) at Fz. D, a similar change-N1 was elicited when the subject ignored (blue) and attended (pink) the sound. E, four consecutive recordings of 200 trials when the subject watched a movie and ignored the stimulus. F, effects of the abrupt change on change-N1. When a 500-ms sound at 70 dB and a 500-ms sound at 75 dB were presented at an even probability (b), change-N1 was not elicited, which clearly contrasts with the upper trace (a) when the abruptly changing deviant was used. Arrowheads indicate the change onset.

Creating an abruptly changing sound stimulus without distorting the sound waveform at the transition is difficult. In the present study, we used a train of brief tone pulses [68] (Fig. 5A). The tone was 800 Hz in frequency and 25 ms in length (5 ms rise and fall times). By using a train of brief standard tones followed by physically different tones, we could easily create an abruptly changing tone stimulus without any undesired edge. In this paper, we refer to the train of standard tones as the Standard stimulus and the train containing physically different tones (deviant) as the Deviant stimulus. For example in Experiment 3 for the detection of the change in sound frequency, the Standard stimulus was a train of 20 brief tones (500 ms in total duration) 800 Hz in frequency, while the Deviant stimulus was a train of ten tones of 800 Hz followed by ten tones of a different frequency.

### Recordings

Evoked potentials were recorded in Experiments 1 ~ 3 for all of the seven subjects. An exploring electrode was placed at Fz referenced to the linked mastoids (P9-P10) of the 10-10 system, since the main component at around 100 ms shows a maximum amplitude at Fz (negativity), and a positive counterpart at P9 and P10 [69]. The mastoid reference results in a maximal amplitude of the change response [56,70] and improves the S/N ratio. Fig. 5B shows an example of recordings in a preliminary study comparing the nose reference and mastoid reference. Fig. 5C shows the distribution of magnetic fields evoked by an abrupt change of sound consistent with symmetric dipoles with an intracellular current directed to the mastoid. Such a field distribution is consistent with that of MMN in previous studies [16-18,20,58]. A pair of electrodes placed on the supra- and infra-orbit of the right eye was used for recording the electro-oculogram. The impedance for all the electrodes was under 5 kΩ. The responses were recorded with a 0.5-100 Hz bandpass filter at a sampling rate of 1000 Hz. The period of analysis was from at least 100 ms before to 350 ms after the onset of deviant sounds. In each experiment, 200 trials without artifacts were averaged for both the Standard and Deviant stimuli. After each waveform was obtained, a difference waveform was calculated by subtracting the waveform for the Standard stimulus from that for the Deviant stimulus. Then the difference waveform was digitally filtered with a low-pass filter of 30 Hz for subsequent analyses. Since a train of 25-ms tones was used, sharp activity at 40 Hz (stimulus-locked activity probably in the primary auditory cortex) could be problematic for the precise determination of the response latency and amplitude when an appropriate low-pass filter was not used.

### Procedures

The experiments were conducted in a quiet, electrically shielded room. The subjects sat in a chair and watched a silent movie on a screen 1.5 m in front of them throughout the experiments. Fig. 5D shows an example of recordings in a preliminary study comparing change-N1 when subjects paid attention to the stimuli and when subjects watched the movie ignoring the stimuli. It is apparent that the subject's attentional state has little effect on change-N1 like MMN [8], and therefore in the present study, change-N1 was recorded in all the experiments while the subjects watched the movie. Under the conditions, change-N1 was elicited stably with a long time course (Fig. 5E). Fig. 5F shows effects of an abrupt change on change-N1. When a 70 dB sound and a 75 dB sound were presented at an even probability, change-N1 could not be elicited (Fig. 5Fb). This indicates that change-N1 is due not to the louder sound itself but to the abrupt change in sound pressure. Therefore, an automatic memory-comparison process appears necessary to shape the change-N1 component. Sound stimuli were presented binaurally through headphones at 70 dB SPL. Five experiments (Experiment 1, Experiment 2, and three experiments in Experiment 3) were carried out on each subject on different days.

#### Experiment 1

First, effects of the interval between the Standard and Deviant stimuli on change-N1 were examined. The Deviant stimulus was a 500-ms 800 Hz tone at 70 dB (standard sound) followed by a deviant sound of 100 ms at 75 dB. The interval between the standard and deviant sounds was either 1, 10, 100, or 1000 ms. The Standard stimulus was identical to the Deviant stimulus except that a 70 dB sound was used instead of the deviant 75 dB sound (Fig. 2A). The two stimuli were presented at the same probability but randomly with an inter-trial interval (offset-to-onset) of 300 ms. In this and following experiments, evoked potentials of different conditions (for example, four conditions with different intervals in Experiment 1) were recorded in separate sessions. The order of the sessions was randomized among subjects. The grand-averaged, filtered (low-pass at 30 Hz) waveforms across subjects are shown in Fig. 1 and the original difference waveforms (non-filtered) are shown in Figs. 2, 3 and 4.

#### Experiment 2

Second, effects of the duration of the standard sound were examined. The Deviant stimulus consisted of two sounds. The first sound (standard) was an 800 Hz tone at 70 dB with a duration of 25, 100, 500, or 1000 ms, and the second sound was a 100-ms 800 Hz tone at 75 dB. There was no blank between the two sounds. The Standard stimulus was similar to the Deviant stimulus but with a 70 dB sound for the second sound. The two stimuli were presented at an even probability but randomly with an inter-trial interval of 300 ms. The order of the four sessions (four different durations) was randomized among subjects.

#### Experiment 3

Third, effects of the magnitude of the physical difference between the standard and deviant sounds were examined for sound frequency, sound pressure, and sound location. In all three experiments, the Deviant stimulus was a 250-ms 800 Hz tone at 70 dB (standard sound) followed without a blank by a 250-ms deviant sound. The Standard stimulus was a 500-ms 800 Hz sound at 70 dB. For the experiment on frequency change, the deviant sound was 808, 816, 824, 840, or 880 Hz. The deviant sound for the experiment on sound pressure change was 72, 73, 74, 75, or 76 dB. The deviant sound for the experiment on sound location change was created by inserting a blank of 0.1, 0.2, 0.3, 0.4, or 0.5 ms into the sound for one ear, that is, an interaural time delay (ITD) of 0.1 ~ 0.5 ms. All the subjects reported that the sound abruptly moved to the left (or right) on hearing the Deviant stimulus with an ITD of 0.5 ms. The blank was inserted into the left sound for three subjects and into the right sound for four. To confirm that the effect of the ITD on change-N1 is actually due to the phase shift between both ears, we additionally tested an insertion of 1.25 ms silence into the left sound (insertion of a longer silent period but without an ITD) in three subjects. However, this deviant sound did not evoke change-N1 at all in two subjects and evoked a small change-N1 at a longer latency (215 ms) than those for the other five ITD sounds (100 ~ 130 ms) in one subject, which was probably due to gap detection.

The two stimuli (Standard and Deviant) were presented at an even probability randomly with an inter-trial interval of 300 ms. The order of the five sessions (five different Deviant stimuli) was randomized among subjects.

### Analysis

In all the experiments, the amplitude of change-N1 was measured and compared among conditions. The amplitude of change-N1 was determined as the amplitude between the peak of change-N1 and the nearest positive peak at an earlier latency. This procedure minimizes problems due to a baseline shift. Although we considered that the amplitude and latency of N100 and P150 evoked by sound changes basically behave similarly under the experimental conditions in the present study, P150 tended to jitter more than N100 in latency. Therefore, we used N100 in this study.

In each experiment, the behavior of the amplitude and latency of change-N1 against variables of each subject were fitted with linear (y = a_1_x + b_1_), logarithmic (y = a_2_ln (x - b_2_)), power (y = x^a3^), and exponential (y = a_4 _+b_3_exp (-x/t)) functions. However, statistical analyses among models were not done because there were only four or five plots in each experiment.

## Abbreviations

ITD: interaural time delay; MMN: mismatch negativity.

## Authors' contributions

KI contributed to planning the study, data collection and analysis, and drafting the paper. TU, KY, NO, and MN contributed to data collection and analysis. YT contributed to constructing devices. SK contributed to revising the paper. RK contributed to drafting the paper. All authors read and approved the final manuscript.
